# Sequential Clinical Changes Following Autologous Mesenchymal Stem Cell Therapy in a Patient With Concurrent Huntington's Disease and Ankylosing Spondylitis: A Case Report

**DOI:** 10.7759/cureus.109019

**Published:** 2026-05-17

**Authors:** Ruslana Gurskaia

**Affiliations:** 1 Neurology, Kabimed Medical Center, Kyiv, UKR

**Keywords:** ankylosing spondylitis, autologous cell therapy, basdai, basfi, case report, huntington's disease, mesenchymal stem cells, regenerative neurology, uhdrs

## Abstract

Huntington's disease (HD) and ankylosing spondylitis (AS) are genetically and pathophysiologically distinct conditions, and their concurrent management may present a complex clinical challenge. A case is reported of a 35-year-old man with genetically confirmed HD and concurrently diagnosed AS, treated with autologous mesenchymal stem cell (MSC) therapy over three consecutive monthly sessions (50 million MSCs per session, total 150 million administered, intravenous and epidural routes). Validated disease-specific assessments were performed at each visit by the treating neurologist before the cell administration of that day: the Unified Huntington's Disease Rating Scale motor score (UHDRS motor), the Bath Ankylosing Spondylitis Functional Index (BASFI), and the Bath Ankylosing Spondylitis Disease Activity Index (BASDAI). Three serial assessments captured changes from baseline (cumulative dose 0) through cumulative doses of 50 million and 100 million MSCs, after which the third (final) dose was administered without subsequent reassessment. Over the two-month observation period, UHDRS motor score declined from 19 to 2, BASFI from 4.0 to 1.0 (with a transient increase to 8.5 at the second visit), and BASDAI from 2.0 to 1.0. No serious short-term adverse events were observed during the active treatment period. Given the single-patient design, concurrent pharmacotherapy, unblinded assessment, absence of objective biomarkers, and absence of post-third-dose assessment, causal attribution to MSC therapy cannot be established. The observation is presented as hypothesis-generating and warrants further investigation under controlled conditions.

## Introduction

Huntington's disease (HD) is a fatal autosomal-dominant neurodegenerative disorder caused by an expanded CAG trinucleotide repeat in the HTT gene, resulting in progressive motor dysfunction, cognitive decline, and psychiatric disturbance [1]. Clinical onset typically occurs in mid-adulthood. No disease-modifying therapy is currently approved; symptomatic management of chorea includes vesicular monoamine transporter type 2 (VMAT2) inhibitors such as tetrabenazine, deutetrabenazine, and valbenazine, the latter approved by the United States Food and Drug Administration in August 2023 for chorea associated with HD [2]. Ankylosing spondylitis (AS) is a chronic immune-mediated spondyloarthropathy characterised by axial inflammation and progressive functional limitation; inflammatory markers may be elevated in some patients [3]. Standard pharmacological treatment includes non-steroidal anti-inflammatory drugs (NSAIDs) as first-line therapy and, in refractory cases, biological agents targeting tumour necrosis factor-alpha (TNF-alpha) or interleukin-17 (IL-17), with Janus kinase (JAK) inhibitors as a more recent option in selected patients; non-pharmacological care including exercise and physiotherapy is also recommended [3].

The co-occurrence of HD and AS in a single patient is uncommon. A non-systematic literature search of PubMed and Google Scholar (search terms: "Huntington's disease" AND "ankylosing spondylitis" AND "mesenchymal stem cells"; date searched: April 2026) did not identify indexed cases documenting concurrent HD and AS treated with autologous mesenchymal stem cell (MSC) therapy; this should not be interpreted as confirmation of absolute novelty given the limited search scope. Autologous MSCs derived from the bone marrow and adipose tissue have been explored preclinically for both neurodegenerative and inflammatory conditions, with proposed mechanisms involving paracrine signalling, neurotrophic factor secretion, and immunomodulation [4-6]. These mechanisms remain hypothetical in the clinical context and have not been confirmed as the basis for clinical efficacy in either HD or AS. Sequential clinical observations following autologous MSC administration in a patient carrying both diagnoses are presented here, with serial assessments using validated disease-specific instruments.

## Case presentation

Patient history and diagnostic confirmation

A 35-year-old man was referred for neurological consultation in October 2017. The patient's father had been diagnosed with HD, and the patient was aware of his genetic risk. Genetic testing for HTT CAG repeat expansion was performed and confirmed pathological repeat length consistent with HD; the exact CAG repeat number was not available in the records reviewed for this report and represents a limitation of the diagnostic detail provided. Molecular confirmation was complemented by characteristic motor signs documented on clinical examination. Concurrently, AS was diagnosed by a consulting rheumatologist according to the Assessment of SpondyloArthritis International Society (ASAS) criteria, imaging arm [7], based on inflammatory back pain features and sacroiliac joint imaging demonstrating sacroiliitis. HLA-B27 status, C-reactive protein (CRP), and erythrocyte sedimentation rate (ESR) were not documented in the available clinical records and represent a limitation of the diagnostic characterisation. The patient had no prior history of immunosuppressive therapy, biological agents, or disease-modifying antirheumatic drugs (DMARDs) and had not previously received any treatment specifically targeting HD motor symptoms.

Baseline assessment

Baseline assessment was performed on 11 October 2017 by the treating neurologist (single, unblinded assessor for all timepoints). The Unified Huntington's Disease Rating Scale motor score (UHDRS motor) [8] was 19, indicating early-to-moderate motor involvement. The Bath Ankylosing Spondylitis Functional Index (BASFI) [9], scored on the standard 0-10 scale, was 4.0, reflecting mild-to-moderate functional impairment. The Bath Ankylosing Spondylitis Disease Activity Index (BASDAI) [10] was 2.0, indicating low baseline disease activity. Total functional capacity (TFC) and formal cognitive or psychiatric assessments were not performed at baseline and represent additional gaps in clinical characterisation. No neuroimaging, cerebrospinal fluid analysis, or fluid biomarkers, including neurofilament light chain (NfL), CRP, ESR, IL-6, or Ankylosing Spondylitis Disease Activity Score (ASDAS), were obtained at any timepoint, limiting the objective characterisation of disease activity and response.

Treatment protocol

Autologous MSCs were prepared from the patient's bone marrow (~90%) and adipose tissue (~10%) by SmartCell biotechnology (Kyiv, Ukraine). Detailed cell-product specifications were not made available to the treating physician for this report, including passage number, viability, sterility testing, endotoxin testing, formal release criteria, and documentation of fulfilment of International Society for Cellular Therapy minimal MSC criteria [11]; this represents a methodological limitation. The author has no commercial or financial relationship with the cell-production facility. Three-monthly treatment sessions were administered: 11 October 2017, 09 November 2017, and 14 December 2017. Each session involved 50 million MSCs: 25 million intravenously (IV) in 100 mL Ringer's solution and 25 million epidurally with lidocaine 0.5% (25 mL). Outcome assessments were performed at each visit prior to that visit's cell administration; consequently, recorded scores reflect the cumulative dose received at each preceding visit (0, 50 million, and 100 million MSCs, respectively), and the effect of the third dose (50 million MSCs administered after the final assessment on 14 December 2017) was not captured by formal reassessment. Plasmapheresis was performed before each session as part of the treatment protocol established by the cell-production facility, with the stated rationale of reducing circulating inflammatory mediators prior to cell administration; a standardised plasmapheresis protocol description was not available to the author, and its potential independent contribution to clinical changes cannot be excluded.

The epidural administration component followed a standard aseptic technique under local anaesthesia. Epidural delivery of MSCs is a non-standard route in HD and has been principally explored in spinal cord injury models [12]; its mechanistic relevance to a primarily striatal neurodegenerative disorder is uncertain, and this delivery approach should be regarded as exploratory rather than evidence-based for HD. No procedural complications, neurological deterioration, or signs of meningeal irritation were observed during or after each administration.

Concomitant pharmacological management throughout the treatment period included the following: ipidacrine (one tablet three times daily for one month, initiated at month 1); thiamine and ascorbic acid combination (300 mg once daily for two months); pramipexole (dopamine agonist, initiated at the start of month 2, after the second MSC administration on 09 November 2017, at 150 mcg daily for 20 days, then 75 mcg daily for 20 days, and then 75 mcg every other day for 20 days); etoricoxib 90 mg once daily for seven days (short-course NSAID, administered at month 2); tizanidine (muscle relaxant) 2 mg daily for 20 days; and retinol/tocopherol supplementation (one capsule daily for 30 days). No immunosuppressive agents, DMARDs, or biological therapies were administered during the observation period. Pramipexole, etoricoxib, and tizanidine each have plausible independent effects on the outcome measures used (motor function, axial pain and function, and stiffness, respectively) and represent meaningful confounders for any attribution of outcome change to MSC therapy.

Outcomes

Serial assessments using UHDRS motor, BASFI, and BASDAI were performed at each monthly visit by the same treating neurologist (Table 1). All assessments were unblinded, and no inter-rater reliability could be determined as a single assessor was used. Results recorded in routine clinical documentation reflect both objective examination components (e.g., UHDRS motor items) and patient-reported components (BASFI and BASDAI are predominantly patient-reported instruments). Adverse-event monitoring was performed through clinical interview and examination at each scheduled visit during the active treatment period (October-December 2017); no formal structured adverse-event reporting tool was used, and no minor adverse events were recorded in the clinical documentation. Follow-up beyond the two-month active treatment period was not available (Table 2).

**Table 1:** Clinical timeline (CARE format) HD: Huntington's disease; AS: ankylosing spondylitis; MSC: mesenchymal stem cell; CARE: CAse REports

Date	Event/assessment	Intervention/medication
Pre-October 2017	HD genetic confirmation; AS diagnosis by rheumatologist	No prior immunomodulatory or HD-targeted therapy
11 October 2017	Visit 1: baseline assessment (cumulative dose: 0 MSCs)	Plasmapheresis + first MSC dose (50M); ipidacrine, thiamine/ascorbic acid started
09 November 2017	Visit 2: assessment after 50M cumulative MSCs (pre-dose)	Plasmapheresis + second MSC dose (50M); pramipexole initiated; etoricoxib 7-day course; tizanidine; retinol/tocopherol
14 December 2017	Visit 3: assessment after 100M cumulative MSCs (pre-dose); final available assessment	Plasmapheresis + third MSC dose (50M, no subsequent reassessment)
Post-December 2017	No further structured follow-up assessments available	-

**Table 2:** Sequential clinical assessments across three timepoints All assessments were performed by the treating neurologist before the cell administration of that visit; therefore, values reflect the cumulative dose received at preceding visits. The third (final) MSC dose, administered after the Visit 3 assessment, was not followed by formal reassessment. UHDRS motor: range 0-124 (higher scores indicate greater motor impairment); BASFI: range 0-10 (established clinically meaningful change ≥1.0 unit); BASDAI: range 0-10 (values <4.0 generally indicate low disease activity and ≥4.0 active disease). All assessments performed by a single unblinded assessor; no inter-rater reliability data was available. The transient increase in BASFI from baseline (4.0) to 8.5 at Visit 2 before declining to 1.0 at Visit 3 is unexplained by the available data and may reflect score variability, post-procedural symptoms, or patient-reported function fluctuation. UHDRS: Unified Huntington's Disease Rating Scale; BASFI: Bath Ankylosing Spondylitis Functional Index; BASDAI: Bath Ankylosing Spondylitis Disease Activity Index; MSC: mesenchymal stem cell

Timepoint	UHDRS motor (0-124)	BASFI (0-10)	BASDAI (0-10)
Visit 1: baseline (11 October 2017); cumulative MSCs: 0	19	4.0	2.0
Visit 2 (09 November 2017); cumulative MSCs: 50M	8	8.5	2.2
Visit 3 (14 December 2017); cumulative MSCs: 100M	2	1.0	1.0

## Discussion

This case report describes the sequential clinical changes across UHDRS motor, BASFI, and BASDAI in a patient with concurrent HD and AS over a two-month period during which autologous MSC therapy and several concomitant medications were administered. The temporal direction of change in UHDRS motor (19 to 2) and the final BASFI value (1.0) are clinically notable, but the broader applicability of this single observation is limited by the unique dual-disease context, the absence of a control condition, the unblinded single-assessor design, the absence of objective biomarkers, and the short follow-up period. The findings should be interpreted as hypothesis-generating rather than as evidence supporting the efficacy of MSC therapy.

Several alternative explanations must be explicitly considered. First, UHDRS motor scores can demonstrate natural variability in early HD, particularly in the period surrounding clinical debut [1]; spontaneous fluctuation, measurement variability, rater effects, and the unblinded nature of the assessment all represent meaningful sources of uncertainty in interpreting the observed changes. Second, pramipexole, a dopamine agonist initiated at the beginning of month 2, after the second MSC administration, has independent effects on motor function and may have contributed to UHDRS motor improvement; controlled HD studies of pramipexole have not demonstrated dramatic motor reversal, but its contribution to the present observation cannot be quantified [13]. Third, etoricoxib (NSAID) is a meaningful confounder for AS outcomes: BASFI is a patient-reported functional index, and pain relief from NSAIDs can produce functional improvement on BASFI even after a brief course; tizanidine, a muscle relaxant, may also have influenced BASFI and BASDAI scores. Fourth, plasmapheresis preceding each MSC administration is itself a potentially active intervention with possible independent effects on inflammatory and clinical parameters.

MSCs derived from the bone marrow and adipose tissue have been described in preclinical literature as secreting neurotrophic factors (including brain-derived neurotrophic factor, nerve growth factor, and vascular endothelial growth factor) and exerting immunomodulatory effects on pro-inflammatory cytokines and regulatory T-cell activity [4-6]. These mechanistic descriptions are derived from preclinical and laboratory studies and do not constitute evidence of clinical efficacy in HD or AS. Whether any such mechanism operated in the present case cannot be inferred from the clinical observations and is not claimed here.

The epidural delivery route warrants explicit caution. This route is non-standard for HD and has limited supporting evidence outside spinal cord injury contexts [12]. Its mechanistic relevance to a striatal neurodegenerative disorder is uncertain, and procedural and regulatory considerations (informed consent for off-label cell administration, oversight of cell-product manufacturing standards) should accompany any future use of similar approaches.

Several structural limitations must be explicitly acknowledged. First, the single-patient design prevents generalisation and precludes any causal inference. Second, the lack of objective biomarkers, including neuroimaging, NfL, CRP, ESR, IL-6, and ASDAS, substantially limits mechanistic and disease-activity interpretation, since the observed score changes cannot be corroborated by independent objective measures. The absence of inflammatory markers is particularly relevant given that BASFI and BASDAI are predominantly patient-reported instruments. Third, follow-up was limited to the two-month active treatment period, representing the most critical limitation of this report; the durability of any observed changes is unknown, and short-term motor score changes in HD without long-term follow-up cannot be reliably distinguished from natural fluctuation. Importantly, because each assessment was performed prior to that visit's cell administration, the effect of the third (final) MSC dose was not captured by formal reassessment, and the documented improvements reflect changes after a cumulative dose of 100 million MSCs rather than the full 150 million administered.

Fourth, the relatively low baseline BASDAI (2.0) and modest baseline BASFI (4.0) limit the interpretability of the AS response, since the patient did not have highly active spondylitis at baseline. Fifth, all assessments were performed by a single unblinded assessor (the treating neurologist), introducing potential measurement bias, expectation effects, and learning effects associated with repeated administration of the same instruments. Sixth, detailed cell-product specifications (passage, viability, sterility, endotoxin, identity markers) were not available to the author from the cell-production facility, limiting the methodological transparency required for translational interpretation. Seventh, the diagnostic characterisation of AS is incomplete, as HLA-B27 status and inflammatory markers were not documented in the available clinical records.

This case is presented as a hypothesis-generating clinical observation in a rare dual-disease context, not as evidence of efficacy. To the author's knowledge based on the limited search described above, no prior indexed case documents autologous MSC administration in a patient with concurrent HD and AS. The primary value of this report is to describe the sequential clinical course in this unusual presentation, to acknowledge the substantial confounders and limitations, and to identify the methodological standards that would be required for any future investigation of MSC therapy in similar settings.

## Conclusions

Sequential clinical changes were observed during a two-month period in which a patient with concurrent HD and AS received autologous MSC therapy alongside several concomitant medications. UHDRS motor score, BASFI, and BASDAI all moved towards lower values at the final assessment, although BASFI showed a transient increase at month 1. The final formal assessment was performed before the third MSC administration; therefore, the documented score changes reflect the period after a cumulative dose of 100 million MSCs, not a post-150-million-MSC outcome. No serious short-term adverse events were observed during the active treatment period; this should not be interpreted as a general safety endorsement given the short follow-up, single-patient design, and absence of standardised adverse-event reporting. Causal attribution of these changes to MSC therapy is not established and is rendered uncertain by concurrent pharmacotherapy, plasmapheresis, unblinded assessment, natural disease variability, and the absence of objective biomarkers. This observation is presented as hypothesis-generating only and does not support the clinical use of autologous MSC therapy in HD, AS, or their co-occurrence outside of properly designed prospective studies with standardised protocols, biomarker incorporation, and long-term follow-up.
